# Experiences and Impact of Face Masks Use on Patient Communication Among Portuguese Nursing Students During Clinical Practice in Hospital Setting: A Qualitative Descriptive Study

**DOI:** 10.1155/jonm/7724749

**Published:** 2025-10-17

**Authors:** Blanca Ortiz-Rodriguez, Vanesa Gutiérrez-Puertas, Hélder Jaime Fernandes, Lorena Gutiérrez-Puertas

**Affiliations:** ^1^Department of Hematology, Torrecardenas University Hospital, Almeria, Andalusia, Spain; ^2^Department of Nursing, Physiotherapy and Medicine, Faculty of Health Science, University of Almeria, Almeria, Andalusia, Spain; ^3^Research Group PAIDI-TIC 019 “Electronic Communications and Telemedicine”, University of Almeria, Almeria, Andalusia, Spain; ^4^School of Health, Polytechnic Institute of Bragança, Bragança, Portugal; ^5^Research Group PAIDI-HUM 061 “Experimental and Applied Neuropsychology”, University of Almeria, Almeria, Andalusia, Spain

**Keywords:** clinical setting, effective communication, face masks, nursing student–patient relation, nursing students, qualitative research

## Abstract

**Aim:**

To explore the experiences and perceptions of nursing students during their clinical practice about communication with patients while wearing a face mask.

**Design:**

A descriptive qualitative study was conducted.

**Methods:**

Data were collected via semistructured, in-depth interviews between December 2024 and April 2025. Eighteen nursing students who communicate with patients while wearing a face mask during their clinical practice were recruited using purposive sampling. The interviews were audio-recorded and transcribed verbatim, and ATLAS.ti v.25.0.1 software was used for a thematic analysis of the interview data. The study adhered to the recommendations for reporting qualitative research (Standards for Reporting Qualitative Research).

**Results:**

The analysis of the results revealed three themes: (1) When Communication Becomes a Challenge, (2) Academic and Clinical Environment Issues that Complicate the Communication Process, and (3) Resources and Strategies for Enhancing Effective Communication with Face Mask Use. Participants identified the elements of communication with patients that are affected by the use of masks, the educational limitations that hindered the communication process and their experience during clinical practice, as well as strategies and resources to improve communication with patients using face masks among nursing students.

**Conclusion:**

Participants identified communication elements affected by face mask use that hinder effective communication and the development of the therapeutic relationship with patients, highlighting the need to implement strategies and resources in both academic and clinical settings.

**Implications for Nursing Management:**

The findings of this study may inform the need for nursing managers to develop and implement educational resources and strategies for preceptors to improve effective communication with patients and satisfaction with the clinical learning environment among nursing students.

## 1. Introduction

Communication is a complex process of interaction involving two or more individuals, through which information, thoughts, and emotions are exchanged using both verbal and nonverbal methods, and the environment influences this process [1]. Effective communication between the nurse and the patient is defined as the dialog established between both parties, in which they speak, listen without interruption, ask questions for clarification, express their opinions respectfully, exchange information, and fully understand the message being conveyed [2]. In the clinical setting, effective communication between nurses and patients is essential for establishing a therapeutic relationship, ensuring patient safety, providing quality care, and achieving optimal health outcomes [3, 4]. Effective communication enables patients to manage their emotions, understand information about their illness, reduce uncertainty, increase participation in decision-making, improve treatment adherence, increase social support and satisfaction [5].

The use of face masks in clinical settings is a crucial measure to prevent contact and respiratory exposure to patient waste, droplets, and airborne particles to prevent the spread of disease [6]. In the clinical setting, it is recommended that nurses wear face masks when caring for patients in isolation due to infections that can be transmitted through the respiratory tract, immunocompromised patients, or during procedures involving exposure to bodily fluids [7]. In hospital units such as oncohaematology, intensive care, surgery, emergency rooms, and general medicine, especially during outbreaks of flu or other respiratory diseases, the use of face masks is a common practice [8]. However, the use of face masks during communication with patients can negatively interfere with both verbal and nonverbal communication, making it difficult to understand speech, see mouth movements, and identify facial expressions and emotions [9]. For nurses, wearing a face mask can lead to a deterioration in effective communication with the patient, negatively affecting the therapeutic relationship and data collection, triggering clinical errors that could compromise the patient's life [10, 11]. The use of face masks negatively affects interpersonal connection and the willingness to engage in communication, increasing anxiety and stress among nurses and patients [8]. In patients, impaired effective communication due to the use of face masks can decrease treatment adherence, positive clinical outcomes, and patient satisfaction [12].

For nursing students, effective communication skills with patients are essential for identifying clinical symptoms, ensuring safety, understanding psychological and emotional needs, providing support and information, and improving patient learning [3]. The nursing degree program integrates education in patient communication skills through experiential learning, recognizing its importance in developing patient-centered care and ensuring quality care [13]. However, nursing students show difficulties in effective communication with patients during their clinical practice [14]. Experiences of communication with patients during clinical practice can contribute to a lack of self-confidence and fear of communication with patients if students do not receive appropriate education [15]. Several studies in nursing education have focused on training in patient communication strategies [14, 16]. However, additional challenges such as the use of face masks in communication between nursing students and patients have not been addressed, which may have consequences during their clinical practice.

Effective communication is essential for providing safe, high-quality nursing care. Several studies have identified barriers to communication with patients in clinical settings [5, 17]. The use of face masks by nursing students can constitute a physical barrier to effective verbal and nonverbal communication with patients [18]. However, few studies have addressed the impact of wearing face masks on communication with patients. In fact, to the best of our knowledge, no studies have been conducted to explore the experiences and perceptions of nursing students during the communication process with patients when wearing face masks. The objective of this study was to explore the experiences and perceptions of nursing students during their clinical practice about communication with patients while wearing a face mask, to improve the understanding of this process, identify barriers, and provide strategies to facilitate effective communication between nursing students and patients when wearing face masks.

## 2. The Study

The objective of this study was to explore the experiences and perceptions of nursing students during their clinical practice about communication with patients while wearing a face mask.

## 3. Methods

### 3.1. Design

A qualitative descriptive study was conducted through semistructured interviews to address the following research questions: What are nursing students' experiences and perceptions of wearing face masks when communicating with patients? This qualitative approach aimed to generate in-depth data on a little-understood phenomenon by learning about students' deep perceptions of communicating with patients while wearing face masks and to provide solutions in the clinical and academic fields [19]. Descriptive qualitative methods with thematic analysis were used as they are appropriate for providing descriptions of phenomena with sparse literature [20]. The Standards for Reporting Qualitative Research (SRQR) was used to report this study [21]. The study was conducted from December 2024 to April 2025.

### 3.2. Participants, Recruitment, and Setting

The study was conducted on students enrolled in the Nursing degree at the Polytechnic Institute of Bragança (IPB), Portugal, in the 2024-2025 academic year. Nursing degree in Portugal consists of four academic years (240 credits). Nursing students begin their clinical placements in the second year. To complete the nursing degree, nursing students at the IPB have performed 120 credits in the clinical setting, which represents half of the nursing degree. Nursing students carry out their clinical placements in the three public hospitals of the Bragança district. Bragança Hospital has 137 beds, Mirandela Hospital has 66, and Macedo de Cavaleiros Hospital has 65, all of which are part of the Local Health Unit of the Northeast (ULSNE). Purposive sampling was used to select the participants. The established inclusion criteria were being a nursing degree, have completed at least one practicum course, and wearing a mask during clinical practice when communicating with patients. Being an exchange student was established as an exclusion criterion due to the potential language barrier, which could interfere with the comprehension of communication with patients.

### 3.3. Data Collection

The researchers developed an interview protocol based on a review of scientific literature, encompassing the most relevant topic of interest for investigation. Interviews were conducted with participants who met the inclusion criteria and provided duly completed informed consent forms. The lead investigator invited the students to participate voluntarily via email, indicating the voluntary nature of the study, its objective, and the confidentiality and anonymous treatment of the data. Data collection was carried out through 18 interviews conducted by the principal investigator, who had extensive experience in qualitative research and communication with patients. The interview was guided and continued purposefully until the researcher reached data saturation, meaning that no new themes or insights emerged from the data. Each interview, lasting between 30 and 40 min, was digitally recorded using a digital recorder, and data confidentiality was strictly maintained. To ensure the anonymity of the participants in the transcription of the interviews, the letter “P” (participants) was employed along with the participant number. The audio recordings were transcribed verbatim within 24 h of the interviews. The audio recordings and transcription were stored to Google drive, ensuring access only to the researchers who analyses the data. The participants reviewed the transcription from the audio recording before data analysis to ensure accuracy. The illustrative quotations selected for inclusion in the manuscript were translated into English by one bilingual researcher. Then, another bilingual researcher backtranslated them into Portuguese and compared the result with the original transcripts to maintain their accuracy. The data were collected between December 2024 and April 2025.

### 3.4. Data Analysis

The audio-recorded interviews were transcribed verbatim using Microsoft Office Word 365 by two researchers. Subsequently, alphanumeric codes were assigned to each participant (P-X). The data were analyzed using the ATLAS.ti Version 25.0.1 software, following the phases outlined by Braun and Clarke for inductive thematic analysis [22]. First, during the transcription of the interviews, two researchers (corresponding author and last author) familiarized themselves with the data, facilitating initial understanding. The transcripts were read repeatedly, generating initial codes from the dataset that were grouped by meaning and patterns. Initial themes were identified, and a thematic map was developed to establish the relationships between the themes. Subsequently, the themes were developed and reviewed to ensure consistency between the codes and the themes. The preliminary themes were discussed and refined by all members of the research team. Finally, a report was written analyzing the previously selected excerpts and relating them to the research question. A conceptual map was developed that included the main themes illustrating the experiences and perceptions of nursing students in relation to communication with patients while wearing face masks.

### 3.5. Rigor and Reflexivity

The methodology and results of the study were carried out based on the recommendations for qualitative research reporting SRQR [21]. The trustworthiness of this study was ensured by adhering to the Lincoln and Guba criteria [23]. Transferability was addressed by providing a comprehensive account of the methods and data collection, as well as direct quotations in the presentation of the findings. Conformability was attained through the independent analysis of transcripts by the correspondence author and last author, following the guidelines established by Braun and Clarke [22], to ensure the validity and accuracy of the data. The discrepancies related to the study design, data analysis, and conclusions were discussed among the research team members until a consensus was reached [24]. To minimize interviewer bias, the interviews were conducted by the principal investigator, who has no faculty affiliation with IPB. All participants were informed of the voluntary nature of their participation, indicating that they could leave the study at any time and would not exert any influence on their grades. The researchers provided the participants with transcripts to verify the information; the participants expressed their agreement, so no changes were made after verification, thus ensuring confirmability [25]. Regarding transferability, a detailed description of the environment, participants, context, and method was provided, serving as a reference for future studies [24].

### 3.6. Ethical Considerations

This study followed the Declaration of Helsinki [26]. Ethical approval was granted by the Ethics Committee of the University (EFM 227/2022). All participants received oral and written information about the study. Written consent to participate was obtained from all participants. The participants were informed about their right to withdraw their informed consent and could decline to answer any questions that were asked, at any time without retribution. They were assured that pseudonyms would be used in the research report to maintain confidentiality.

## 4. Results

A total of 18 individual interviews with nursing students were conducted from December to April. Overall, 66.70% of the participants (*n* = 12) identified as female and 33.30% as male (*n* = 6). The mean age of the participants was 21.28 (SD = 1.12) years, with a range from 20 to 24 years. Of the total participants, 44.40% (*n* = 8) were in their second year, 27.80% (*n* = 5) in their third year, and 27.80% (*n* = 5) in the fourth year of the nursing degree. Regarding the number of practicums, 44.40% (*n* = 8) of the participants performed two practicums, 27.80% (*n* = 5) performed three practicums, and 27.80% (*n* = 5) performed four practicums of the nursing degree (Table 1).

**Figure 1:**
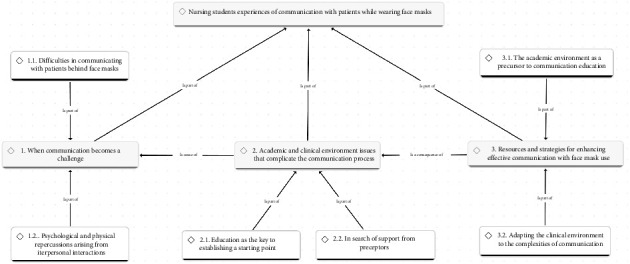
Conceptual map based on nursing students' experiences of communication with patients while wearing face masks.

The three themes that emerged from the data analysis were as follows: (1) When Communication Becomes a Challenge, (2) Academic and Clinical Environment Issues that Complicate the Communication Process, and (3) Resources and Strategies for Enhancing Effective Communication with Face Mask Use. The description of the themes and subthemes in this study is given in the following (Figure 1).

### 4.1. When Communication Becomes a Challenge

This theme describes the participants' experiences in relation to communication with patients while wearing face masks during their clinical practice. This topic identifies the aspects that hinder effective communication, as well as the emotions and symptoms caused by wearing face masks during interaction with patients.

#### 4.1.1. Difficulties in Communicating With Patients Behind Face Masks

One common aspect highlighted by the participants was the inability of the patients to identify their facial expressions when their faces were covered with face masks, limiting nonverbal communication to eye contact.

I feel that my face is empty, the impact of facial expressions is lost, and I am left only in a look, and that is not enough to convey everything I want to express (P-12).

In addition, several of them argued that wearing a face mask interferes with speech intelligibility, making it difficult to convey messages clearly and affecting their ability to provide information, respond to queries, or educate others.

I think that with the face mask, education and information are not conveyed as clearly, and that means they don't understand what is being explained to them properly (P-3).

#### 4.1.2. Psychological and Physical Repercussions Arising From Interpersonal Interactions

Most participants emphasized the impact on their mental and emotional well-being, considering the use of face masks to be an obstacle that prevents effective communication and can put patients at risk. They also mentioned that the difficulty in being identified from one interaction to another, establishing a therapeutic relationship, and the perceived dissatisfaction of patients triggered emotions of frustration, despair, and anger during interactions.

I am worried that, with the mask, they will not understand me and this will generate misunderstandings that endanger the patient (P-7).

What affects me most is that when I enter the room, the patient doesn't recognise me from one day to the next because of the face mask. So how can they trust me? They see me and don't know if it was me who was with them the previous afternoon or if it was someone else. Then, of course, they find it difficult to talk to me again, and that frustrates me, drives me crazy and even makes me angry (P-14).

On the other hand, several participants argued that wearing a face mask reduced the tone and clarity of their voice, forcing them to raise their voice, repeat information, and intensify their gestures. They also emphasized experiencing physical discomfort such as sore throats, headaches and fatigue, which increased during interactions until they felt exhausted.

When I wear a face mask, people often tell me that I speak too softly and that they cannot understand me, so I end up raising my voice and gesturing desperately, repeating the same thing over and over again. I leave the room with a headache and a sore throat, exhausted from the stress of trying to make myself understand (P-15).

### 4.2. Academic and Clinical Environment Issues That Complicate the Communication Process

This theme encompasses students' perceptions of their experiences communicating with patients while wearing face masks, focusing on the impact this had on their clinical practice and their work as future nurses. In this way, participants described the perceived obstacles in both academic and clinical settings that negatively interfered with establishing effective communication with patients and satisfaction with their work during internships due to educational limitations.

#### 4.2.1. Education as the Key to Establishing a Starting Point

Most participants emphasized that they had no specific prior knowledge about communicating with patients while wearing face masks and did not know how to act, which caused them anxiety and stress due to their inability to cope with the communication challenges arising from wearing a face mask. In addition, some participants commented that, despite having some prior knowledge of patient communication, the use of face masks requires specific training to improve their self-efficacy and clinical performance, as well as effective communication during interactions with patients.

I feel like I'm not prepared to communicate with a face mask on. I freeze up, get nervous, and feel overwhelmed. If I knew how to handle it (communication with a face mask) better, I would probably feel less stress and anxiety (P-2).

In class, we have done some exercises to improve communication with patients, and in general I have felt capable and able to manage the situation well, but we haven't worked at all on communicating while wearing a face mask, and I think that's essential (P-4).

#### 4.2.2. In Search of Support From Preceptors

On the other hand, some participants expressed a lack of availability of preceptors to provide them with support, adequate training, and guidance during their interactions with patients due to high workloads and the number of nursing students they were mentoring, perceiving themselves to be a burden on their preceptors.

My preceptor sent us out alone to inform and explain. He seemed overwhelmed, perhaps because there were too many students and too much work. That's why he couldn't help us improve the way we communicated with our face masks on (P-5).

In contrast, some participants reported complaints from preceptors if they spent too much time with patients. In addition, some participants reported reactions of surprise, anger, and sarcasm from the preceptors if they asked questions related to communication with face masks. This rude attitude from the preceptors led some students to feel demotivated, ambiguous about their continuity in the nursing field, and unwilling to work at that hospital in the future.

You couldn't take long with the patient because some tutors got upset, and you couldn't ask your questions about how to communicate with the mask, since they responded with surprise, irony or anger, which demotivated me, made me doubt about continuing with this profession and took away my desire to return to that hospital (P-16).

### 4.3. Resources and Strategies for Enhancing Effective Communication With Face Mask Use

This theme identifies different resources and strategies to be implemented in academic and clinical institutions that participants consider essential for developing effective communication, establishing a relationship of trust with patients, and ensuring satisfaction with clinical practices when faced with challenges such as the use of face masks in interactions with patients in the clinical setting.

#### 4.3.1. The Academic Environment as a Precursor to Communication Education

Most participants considered the inclusion of specific content on patient-centered communication with the use of face masks in nursing degrees to be essential for improving their effective communication with patients. In addition, some participants argued that prior exposure to these situations in a simulated environment can have important implications for their experiences of communication with patients during their internships, as it allows them to improve their self-confidence and self-efficacy.

I think specific preparation should be provided to learn how to communicate while wearing a face mask, as masks greatly limit expression and comprehension (P-1).

For me, simulation would be very helpful in learning how to manage these types of situations safely, effectively, and with greater self-confidence (P-13).

Participants emphasized the importance of implementing psychological support in educational institutions that promote mental well-being and resilience in order to develop adequate coping skills for stressful situations, such as communicating with patients while wearing a face mask. Some participants acknowledged that receiving external psychological support helped them cope with the challenges posed by wearing a face mask.

In my experience, psychological support has helped me cope better with difficult moments, and I think communicating with a face mask can be one of those moments. Therefore I believe it is essential to have psychological support, as we face many stressful situations like this daily (P-10).

#### 4.3.2. Adapting the Clinical Environment to the Complexities of Communication

Participants felt that clinical institutions should include resources during interactions, such as the use of photo cards or transparent face masks, thus facilitating the identification of both facial expressions and students. In addition, some participants indicated that the use of electronic devices such as tablets to enable communication through virtual meeting, before the first face-to-face meeting and as a communication support tool, would improve the establishment of the therapeutic relationship, empathy, and satisfaction with care.

‘I think that, even if it is the first time we are talking to the patient, resources such as tablets could be used to allow us to see each other's faces. In addition, aids such as cards, photos or even transparent face masks would greatly improve communication and the relationship with the patient' (P-5).

Several participants stressed the importance of developing and implementing reception protocols that include information on available resources and strategies to facilitate communication with patients when wearing face masks. Participants argued that these strategies would enable more effective communication with patients, contribute to improving their training during internships, and increase their satisfaction with clinical practice and the hospital environment.

‘There may be resources available, but without a reception protocol or someone to explain it to you, how are you going to know they are there? It's a shame, because in the end you end up with poor communication with the patient and you leave the internship with a bad taste in your mouth, knowing that you could have done better' (P-6).

In contrast, some participants emphasized the need to educate preceptors in patient communication skills while wearing face masks, as well as further training in mentoring students. This would address the patient communication skills training needs of nursing students.

‘I think preceptors should also be educated on how to communicate with a face mask, because behind that barrier there is a person.' In addition, I think it would be very useful for them to be prepared to support those who are learning' (P-9).

## 5. Discussion

The aim of this study was to explore the experiences and perceptions of nursing students during their clinical practice about communication with patients while wearing a face mask. In line with previous studies on communication with patients while wearing face masks [8, 11, 27], our findings provide new information on how nursing students experience wearing face masks when communicating with patients in clinical settings and the resources and strategies needed to overcome the challenges identified. The nursing students' experiences of communicating with patients were negatively affected by the use of face masks, which interfered with both verbal and nonverbal communication, hindering effective communication. Consistent with the data from this study, the use of face masks makes it difficult to identify emotions and reduces speech comprehension [8, 27]. Facial expressions are an essential element in nonverbal communication with patients to show emotional expression and an empathetic attitude, facilitating effective communication with patients [9]. Previous studies have shown that the use of face masks muffles speech sounds, altering vocal projection and reducing the intelligibility of the message conveyed, which is exacerbated in hospital settings with high ambient noise [6, 28].

The findings of this study highlight communication barriers generated by the use of face masks that hinder recognition, the establishment of a therapeutic relationship, and patient satisfaction with care, affecting the mental and emotional well-being of nursing students, coinciding with data reported in a recent study conducted on nurses [11]. Previous studies have shown that barriers to communication with patients in the clinical setting affect health outcomes, quality of care, patient safety, and patient satisfaction with care [5, 17]. In line with the findings of this study, the use of face masks during interactions with patients can cause a sense of loss of professional and personal identity by concealing distinctive facial features that facilitate recognition, negatively impacting the establishment of a therapeutic relationship [28]. Similarly, the mechanisms developed to compensate for the communication difficulties caused by wearing a face mask can trigger physical discomfort such as headaches, sore throats, and fatigue. This may be due to the prolonged use of the face mask during the working day, which has been associated with increased physical and mental exhaustion, as well as reduced clinical performance [29, 30].

Furthermore, the lack of specific education on communication skills with patients when wearing face masks received during nursing degrees and the difficulty in coping with stressful situations such as those generated by their use limited the self-efficacy and clinical performance of nursing students. Recently, nursing education lacks standardization in patient communication training within the basic curriculum and is integrated into multiple subjects [31]. This fragmented approach could explain why patient communication problems are a challenge for nursing students when transitioning to clinical practice [31, 32]. Consistent with the findings of the present study, the positive association between self-efficacy and academic performance among nursing students is well established in the literature [33, 34]. Nursing students with higher self-efficacy show better learning outcomes and are more likely to accept challenging clinical tasks [33]. In addition, the development of resilience among nursing students can contribute to increased self-efficacy and coping with stressful situations in the clinical setting [35]. Therefore, it is necessary to develop interventions that promote resilience and self-efficacy among nursing students, as these could improve effective communication with patients in stressful situations such as those generated by the use of face masks.

Similarly, the study findings reveal that the lack of support from preceptors was an obstacle in the process of communicating with patients, negatively affecting their satisfaction with the practices and the clinical environment. Several studies have highlighted that the excessive workload and multiple responsibilities of preceptors reduce their ability to provide effective support to students, compromising crucial processes such as guidance, supervision, and resolution of clinical doubts, which can trigger frustration, ambiguity in clinical learning, and demotivation in nursing students [36, 37]. The lack of preparation of students, the expectations of preceptors regarding students, as well as work overload can lead to frustration and rude attitudes in preceptors toward students [38], as revealed by the findings of this study. On the contrary, the positive attitude of preceptors helps to increase students' satisfaction with the clinical environment and their intention to remain in the hospital as future nurses [39, 40]. It is necessary to reinforce support for preceptors and reduce their concerns about other issues such as work overload so that they can guide future nurses.

The findings of this research revealed the need to implement strategies and resources in both academic and clinical settings to improve effective communication and establish a therapeutic relationship with patients, as well as to increase mental well-being and satisfaction with practices. In relation to the academic environment, specific theoretical and practical training in communication skills with patients wearing face masks is essential to develop effective communication and facilitate the transition to the clinical environment. In line with the data from this study, specific education in soft skills through simulation improves the self-efficacy and clinical performance of nursing students [41, 42]. In addition, previous learning experiences in patient communication can increase nursing students' self-confidence during their interactions with patients in the clinical setting [9]. Another strategy identified to improve nursing students' transition to clinical practice and coping with stressful situations was the development of psychological support programs that promote resilience. In this regard, the academic environment is crucial in helping nursing students process stressful situations or emotions that arise during clinical rotations and make sense of their experiences, which can promote the development of resilience and healthy coping behaviors [43, 44]. Therefore, nursing educators need to keep the nursing degree curriculum up to date and implement strategies aimed at enhancing resilience and encouraging nursing students to adopt positive coping strategies to improve learning and satisfaction during clinical practice.

The development of effective communication with patients wearing face masks in clinical settings requires the support of nursing managers to implement resources and strategies that help overcome communication barriers, enable the establishment of a therapeutic relationship, and ensure patient satisfaction, as previous studies have shown [8, 27]. The students suggested incorporating resources such as photographs on uniforms and the use of transparent face masks or tablets to facilitate identification and enable the development of a therapeutic relationship with the patient. The use of photographs on uniforms can help humanize care and facilitate the development of a therapeutic relationship with the patient [8, 45]. The use of transparent face masks increases the feeling of trust as it helps to see the whole face, facilitates lip reading, and identifies the professional [27, 28]. Similarly, the implementation of new technologies to complement face-to-face care with virtual care was shown to facilitate continuity of care, participation in decision-making, and patient satisfaction with the care received during the pandemic restrictions [46]. On the other hand, the development and implementation of reception protocols for nursing students, as well as the training of preceptors in communication skills with the use of face masks and student tutoring, would improve self-efficacy, clinical performance, and satisfaction with the practices. The lack of guidelines to follow to overcome the obstacles generated by the use of face masks increases the time needed for care and the workload, highlighting the need to educate nurses in compensatory communication strategies [8, 27]. In addition, preceptors are not prepared to provide adequate mentoring; their expectations of students are often excessive and not adapted to their mentoring role, highlighting the need to provide specific training to preceptors [39, 40]. Mentoring preceptors is crucial to reducing the intention to leave the hospital and improving satisfaction with the clinical learning environment [47]. Therefore, nursing managers need to implement specific training programs so that preceptors acquire the necessary skills to preceptors' students and ensure adequate, up-to-date, and evidence-based education [40].

### 5.1. Limitations and Future Research

Some limitations should be considered in this study. The participants had completed their internships in hospitals in the same country, which could limit the cultural transferability of the findings. A possible limitation characteristic of the nature of qualitative research is that the data were collected through interviews, which could induce social desirability bias, as participants may have provided answers they considered socially acceptable and did not accurately reflect their experiences and perceptions. The study explored the perceptions of nursing students and excluded nurses, other students, and health professionals. A heterogeneous sample of students and health professionals would have provided different perspectives. Similarly, the sample selection did not consider the experiences and perceptions of patients and their families regarding communication with students or other professionals wearing face masks. Future studies could develop qualitative research that includes nurses, other healthcare professionals, and nursing managers to gain a deeper insight into this phenomenon. It would also be interesting to develop qualitative studies on the experiences and perspectives of patients and family members on communication with nursing professionals to identify perceived barriers to communication and strategies to ensure effective communication, which is essential for quality care. In addition, studies are needed to explore the effectiveness of strategies that increase effective communication between nursing students and patients when wearing face masks, in order to include content in nursing curricula that ensures adequate education to better prepare future nurses.

### 5.2. Implications for Nursing Management

This study emphasizes that the use of face masks negatively affects nursing students' effective communication with patients, interfering with their relationship with patients and their clinical experience during clinical practice. Nursing managers must be aware and recognize that the use of face masks in the clinical setting is one of the complexities faced by students and professionals working in units with immunocompromised patients or patients with contagious respiratory diseases. This study highlights the need to develop educational programs on communication skills using face masks for both nursing students and preceptors. In addition, nursing managers can implement training programs for preceptors who mentor nursing students to address the complexities these students face in order to improve academic and clinical training during their placements. Likewise, educational institutions should implement strategies that foster resilience and the ability of students to cope with stressful situations to prevent them from leaving the profession and increase their desire to remain in the hospitals where they carry out their clinical practice. In addition, nursing managers can implement educational programs for preceptors who preceptors nursing students to ensure adequate learning and student satisfaction during their placements. Similarly, it would be beneficial to develop induction protocols for nursing students that include available resources and communication strategies to overcome the barriers associated with wearing face masks.

## 6. Conclusion

This study provides valuable information on the experiences and perspectives of nursing students regarding communication with patients while wearing face masks. The results identified the elements of verbal and nonverbal communication that are affected by the use of face masks, hindering effective communication and the development of a therapeutic relationship with the patient. Educational limitations in both academic and clinical settings impact the process of communicating with patients, satisfaction with practical training, and future work as nursing professionals. The participants highlighted the need to implement strategies and resources such as updating the nursing degree curriculum in communication skills with patients when wearing face masks, developing interventions that promote resilience, and incorporating resources and specific education for preceptors that could improve effective communication with patients and satisfaction with practical training among nursing students. These findings provide an opportunity to reflect on the importance of ensuring effective communication with patients when wearing face masks among nursing students by guaranteeing adequate education that facilitates the transition from theory to practice and increases their intention to stay in the environment in which they carry out their internships.

## Figures and Tables

**Table 1:** Sociodemographic characteristics of participants.

Code	Sex	Age	Year enrolled in	Practicums (number)
P-1	Female	20	3rd year	Three
P-2	Female	21	2nd year	Two
P-3	Male	20	2nd year	Two
P-4	Male	20	3rd year	Three
P-5	Female	21	4th year	Four
P-6	Female	21	2nd year	Two
P-7	Male	21	2nd year	Two
P-8	Female	22	4th year	Four
P-9	Female	22	4th year	Four
P-10	Female	20	2nd year	Two
P-11	Female	20	3rd year	Three
P-12	Female	22	2nd year	Two
P-13	Male	23	2nd year	Two
P-14	Female	22	4th year	Four
P-15	Female	21	2nd year	Two
P-16	Male	21	3rd year	Three
P-17	Male	24	4th year	Four
P-18	Female	22	3rd year	Three

## Data Availability

The data supporting this research are available from the corresponding author upon reasonable request.
